# Altered Basal Ganglia Network Topology Associated With Auditory–Motor Synchronization

**DOI:** 10.1002/brb3.70695

**Published:** 2025-08-27

**Authors:** Stéphanie K. Lavigne, Jonathan H. Burdette, Mohsen Bahrami, Paul J. Laurienti, Robert G. Lyday, Michael H. Thaut

**Affiliations:** ^1^ Music and Health Science Research Collaboratory, Faculty of Music University of Toronto Toronto Ontario Canada; ^2^ Laboratory for Complex Brain Networks, Department of Radiology Wake Forest University School of Medicine Winston‐Salem North Carolina USA; ^3^ Collaborative Program in Neuroscience, Faculty of Medicine University of Toronto Toronto Ontario Canada

**Keywords:** auditory–motor synchronization, basal ganglia network, BOLD fMRI, brain connectivity, brain networks, graph theory

## Abstract

**Introduction:**

Auditory–motor synchronization (AMS) embedded in Rhythmic Auditory Stimulation (RAS) is a validated method to improve gait, upper limb function, and motor speech in people with neurologic disorders like Parkinson's disease (PD). Predictable auditory cues optimize spatial movement patterns, and research has suggested that AMS reduces the brain's reliance on dopaminergic (DA) response in the ventral striatum. To gain a mechanistic understanding of the positive clinical outcomes related to AMS, this pilot study investigates the effects of AMS on the basal ganglia network (BGN) using brain network science methods.

**Methods:**

Fourteen healthy adults (aged 22–37, seven females) completed two fMRI finger tapping tasks, a self‐paced continuation (self) task and an auditory–motor synchronized (sync) task, both performed at 1 Hz. Using a modularity analysis of brain network data, we assessed the spatial consistency of the BGN. Additionally, we used a mixed‐effects regression framework to test the hypotheses that changes in global and local efficiency are associated with the experimental tasks.

**Results:**

The spatial consistency of the BGN community was significantly greater in the sync task compared to the self task. Then, the regression model showed a significant change in the BGN's efficiency in the sync task over the self task. Specifically, the probability and the strength of connections between highly efficient nodes were significantly greater, indicating a more synchronized BGN.

**Conclusion:**

AMS significantly changed the network topology of the BGN compared to no AMS. Specifically, the BGN became more functionally synchronized with AMS due to, mainly, greater network efficiency. These findings contribute to the growing mechanistic knowledge of how the BGN functional connections change with AMS and why AMS is a powerful tool to treat neurologic disorders such as PD.

## Graphical abstract

Finger tapping to 1‐Hz tones significantly changed the topology of the basal ganglia network (BGN) in a group of healthy young adults. Specifically, the BGN became more functionally synchronized due to, mainly, greater network efficiency during the 1‐Hz tone synchronized task, compared to the self‐paced task.

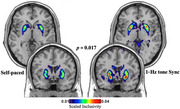

## 1 Introduction

Mapping periodic motor patterns, such as gait, to auditory beats is an optimization method employed in music‐based neurorehabilitation. Rehabilitation specialists use the standardized technique Rhythmic Auditory Stimulation (RAS; Hoemberg and Thaut 2014) from Neurologic Music Therapy that capitalizes on auditory–motor synchronization (AMS) to retrain and re‐educate gait, upper limbs, and motor speech in individuals recovering from a stroke (Malcolm et al. 2015; Teasell et al. 2020), traumatic brain injury (Hurt et al. 1998), spinal cord injury (de lʼEtoile 2008; Alashram et al. 2019), Parkinson's disease (PD) (McIntosh et al. 1997; Ghai, Ghai, Schmitz et al. 2018; Braunlich et al. 2019; Braun et al. 2019), multiple sclerosis (Ghai and Ghai 2018), cerebral palsy (Kim et al. 2011; Ghai, Ghai, and Effenberg 2018), autism spectrum disorder (Bharathi et al. 2019), and other disorders of the nervous system (Thaut and Abiru 2010; Thaut et al. 2015). RAS is also used in sports training and performance (Shaffert et al. 2019). Thus, practitioners of music‐based therapies find rhythm at the core of research efforts on the brain and music (Thaut and Abiru 2010; Thaut et al. 2015; Braun et al. 2022), although the exact mechanism underlying this successful therapy needs further investigation.

There is growing evidence that the basal ganglia (BG) are key brain components involved in the success of AMS techniques. The BG represent a group of deep gray structures in the brain that are involved in motor control and learning, executive functions, and emergent behaviors. The BG nuclei receive projections from nearly all regions of the neocortex, the thalamus, the brainstem, and limbic structures. The BG are further associated with initiation and termination of movements (Kandel et al. 2021). Ongoing research is uncovering the relationship of dopaminergic (DA) responses to RAS in the BG. Koshimori et al. (2019) investigated the DA responses in healthy young adults as they performed a right‐hand finger tapping task with and without RAS. Using positron emission tomography (PET) and the radioligand [^11^C]‐(+)‐PHNO‐PET that detected D3 dopamine receptors within the ventral striatum and the globus pallidus, they demonstrated lower DA response in the left ventral striatum with RAS. These results provided initial insights into neurochemical mechanisms underlying rhythmic cueing in the BG.

AMS embedded in RAS is an especially useful rehabilitation tool in PD. PD affects the BG with decreased dopamine leading to BG dysfunction. A resting‐state fMRI connectivity analysis of the BG network showed that PD patients “off” DA medication had reduced connectivity within BG clusters, whereas those “on” medication had BG connectivity more similar to healthy controls (Szewczyk‐Krolikowski et al. 2014). Clearly, an optimally functioning BG improves the physical symptoms of PD. Our study did not recruit PD patients yet, as we believe that changes in BG network characteristics based on a motor task synchronized to a tone must first be investigated in healthy brains.

The aim of our study was to investigate the functional connectivity of AMS in healthy young adult brains as a baseline for future inquiries into the brain mechanisms underlying AMS in the PD population. To our knowledge, brain functional network methods have not yet been applied to the study of AMS. We used two simple finger tapping paradigms: self‐paced and synchronized with isochronous tones. Given the known improvement in motor function during AMS, we hypothesized that AMS would improve the intrinsic connectivity of the basal ganglia network (BGN), reflected by alterations in global and local connectivity patterns, while also leading to better tapping performance.

## 2 Materials and Methods

### 2.1 Dataset

#### 2.1.1 Participants

Eighteen healthy young adults were enrolled in our study (ages 18–38 years old). Inclusion criteria consisted of being a nonmusician (defined as having received less than 3 years of formal musical training, including lessons and/or organized ensemble playing for instrumentalists or vocalists) and being right‐handed according to the Edinburgh Handedness Inventory (EHI) questionnaire. Exclusion criteria consisted of having an active neurological or psychiatric disorder (individuals diagnosed with depression but on stable medical treatment for at least 4 weeks were not excluded), failing the hearing test based on the bilateral finger rubbing test, color blindness, pregnancy, and a history of a traumatic brain injury. Individuals with the potential to be cognitively or psychologically impaired or who otherwise had a limited capacity to provide consent were asked not to participate.

#### 2.1.2 Recruitment

Participants were recruited through the Laboratory for Complex Brain Networks database, via word‐of‐mouth, or through advertisements posted around Wake Forest University School of Medicine (WFUSM) in North Carolina.

### 2.2 Final Study Cohort

Of the initial consented 18 participants, two were not scanned as they wore permanent retainers, one participant withdrew consent before the MRI scan due to concerns that earbud tones could cause eardrum damage, and one participant was removed due to instrument capture errors in their tapping data. The final sample (*N* = 14) was composed of seven females and seven males aged between 22 and 37 years old (*M* = 30.4, *SD* = 5.0). Table 1 shows the participant demographics. Two participants had a history of a depression and one of an anxiety disorder, and as their condition had resolved, they no longer required medication. They all completed university‐level education, with the highest level completed being a bachelor's degree (*n* = 4), a master's degree (*n* = 7), or a PhD degree (*n* = 3). Participants were right‐handed, and their right‐handedness scores based on the EHI questionnaire ranged between 80% and 100% with an average score of 90% and a median of 100%. Participants were all nonsmokers. Regarding alcohol consumption, three participants had no drinking history, and all other participants had minimal drinking history, with on average 1.6 drinks per week (*SD* = 1.7) for our sample. No participants meet NIH requirement for moderate drinking (USDA and HHS 2020).

**TABLE 1:** Demographic information of our sample (*N* = 14) of healthy young adults.

Age	Frequency	%
18–24	3	21
25–29	1	7
30–34	6	43
35–38	4	29
Sex		
Female	7	50
Male	7	50
Race		
Caucasian	8	57
African American	4	29
Asian	1	7
Caribbean	1	7
Hispanic ethnicity		
Yes	2	14
No	12	86
Right‐handedness		
Yes	14	100
No	0	0
Years of private music instruction		
0	10	71
1	2	14
2	1	7
3	1	7
4+	0	0
Highest level of education completed		
Bachelor	4	4
Master	7	50
PhD	3	21
Smoking		
Yes	0	0
No	14	100

### 2.3 Protocol

The study protocol was reviewed and approved by the WFUSM IRB. Participants attended two in‐person sessions, a medical screening session, and a magnetic resonance imaging (MRI) scanning session. Participants were financially compensated for taking part in this study.

#### 2.3.1 Medical Screening Session

Through an initial phone screening, we assessed the medical eligibility of participants. Eligible participants were invited to an in‐person visit where HIPAA‐compliant informed consent was obtained. Screening tests administered included a medical history questionnaire, a musical history questionnaire, the EHI questionnaire, and the bilateral finger rubbing auditory test.

#### 2.3.2 MRI Scanning Session

The MRI scanning session took place at the Center for Biomolecular Imaging at WFUSM, around the same time of the day for every participant. Standard anatomical brain recordings were acquired, as detailed below. Functional MRI (fMRI) brain sequences were performed using a single continuous 6‐min recording period for each task. The network analyses conducted for this study require steady‐state brain recordings.

#### 2.3.3 fMRI Tasks

Tapping tasks used the right index finger. First, participants performed a self‐paced continuation (self) task, followed by an auditory–motor synchronized (sync) task. In the self task, ten 1‐Hz tones (interstimulus interval [ISI] of 1000 ms) were presented to set the pace, after which participants self‐paced their taps until the end of the recording period. In the sync task, participants were instructed to tap their finger in synchrony to the continuously presented 1‐Hz tones for the entire recording period.

### 2.4 Materials and Data Acquisition

#### 2.4.1 MRI Data Acquisition

The MRI data acquisition was performed on a 3T Siemens Magnetom Skyra with a 32‐channel head coil. Anatomical scans used the Magnetization Prepared Rapid Acquisition Gradient Echo (MPRAGE) GRAPPA2 sequence acquired in the sagittal plane (TR/TE = 2300/2.99 ms; flip angle = 9°; voxel dimension = 1 × 1 × 1 mm^3^; 192 slices per volume; slice thickness = 1 mm). fMRI images captured variations in the blood‐oxygenated‐level‐dependent (BOLD) signal and employed a single‐shot gradient echo‐planar imaging (EPI) sequence acquired in the transversal plane (TR/TE = 2000/25 ms; flip angle = 75°; FoV = 224 mm; voxel dimension = 3.5 × 3.5 × 5.0 mm^3^; 35 slices per volume; 177 volumes; slice thickness = 5.0 mm).

### 2.5 Behavioral Material

The finger tapping protocol was implemented in the E‐prime 3.0 software from Psychology Software Tools (https://pstnet.com/products/e‐prime). The auditory stimulus was a 1‐Hz tone (60 ms total duration, 10 ms fadeout, 1000 Hz pitch frequency) delivered via MRI‐compatible earbuds. Tones presented at the 1 Hz rate reflected an ISI of 1000 ms. During the experiment, participants maintained their gaze on a fixation cross. The E‐prime Serial Response Box device captured finger responses as key presses, using a single key with a 0‐ms debounce period. Participants were not provided with auditory feedback of their taps.

#### 2.5.1 Behavioral Data Analysis

We measured performance on our finger tapping tasks using the absolute period error, defined as the absolute time difference between the ISI of 1000 ms, or time interval between two tone presentations, and the interresponse interval (IRI), or time interval between two response taps. Thus, the absolute period error reflected the period relationship between the IRI and the ISI (Thaut, Miller et al. 1998). We averaged the absolute period error for each participant over each task to obtain a measure of tapping accuracy, which served as our measure of tapping performance. A paired sample *t*‐test was used to compare tapping accuracy between tasks. We used the standard deviation of the absolute period error to compare the tapping variability between tasks. Additionally, we examined synchronization error, defined as the time interval between a tone onset and its corresponding tap time, in the sync task only, as tones disappeared after 10 s in the self task as a continuation task. A positive synchronization error value indicates that taps occur after tone onsets, while a negative synchronization error value indicates that taps precede tones (Thaut, Miller et al. 1998).

#### 2.5.2 Data Cleaning

Taps that occurred within the first 10 s of both tasks were removed. Taps with an IRI 50% shorter or longer than the associated ISI were removed. In the case of double tapping, the second tap was removed.

### 2.6 MRI Preprocessing, Brain Network Generation, and Network Analysis

We explored AMS in the brain by analyzing brain networks in two ways: (1) at a voxel‐wise resolution to assess the BGN consistency using a permutation analysis (Hayasaka and Laurienti 2010; Standley et al. 2013) and (2) at a standard atlas‐based resolution to perform a mixed‐model multivariate regression analysis of the BGN (Bahrami et al. 2019; Simpson and Laurienti 2015).

Preprocessing steps were completed with Statistical Parametric Mapping (SPM12, http://www.fil.ion.ucl.ac.uk/spm) unless otherwise stated. This paper features two separate analyses, and, therefore, we had separate preprocessing for each.

### 2.7 Voxel‐Wise Preprocessing

The structural T1 images were coregistered to the MNI‐152 brain Template (Fonov et al. 2011). SPM's unified segmentation split the T1 images into white matter (WM), gray matter (GM), and cerebrospinal fluid (CSF) segments while simultaneously warping the images to the MNI‐152 template. The first 10 volumes of the fMRI time series were removed to allow the BOLD signal to reach a steady state. Then, the fMRI time series for each participant were slice‐time corrected and realigned to the first image of the scan. ICA‐AROMA (Pruim et al. 2015) was performed to correct for motion. Functional images were coregistered to the participant's structural image and then warped to standard space using the previously calculated SPM deformation field. We applied a bandpass filter at 0.009–0.08 Hz to the warped images and regressed out the average signal from three segments (GM, WM, and CSF), as well as the six motion parameters calculated during realignment. Any residual motion artifacts were corrected using the motion scrubbing procedure (Power et al. 2012).

### 2.8 Voxel‐Wise Brain Networks

#### 2.8.1 Network Generation

Voxel‐wise brain networks were generated for each participant by computing the pairwise Pearson's correlation between the time series of each voxel (~20,000) with every other voxel in the warped functional images. We applied a threshold to the resulting connection matrix using the formula *S* = log(*N*) / log(*K*), where *N* represents the total number of network nodes (or voxels) and *K* is the average network degree (*k*, number of links or edges per network node). This threshold application yielded an adjacency matrix representing networks of equivalent node density across the group, thereby allowing their comparisons (Rubinov and Sporns 2010). We used *S* = 2.5 based on previous work on network fragmentation (Telesford et al. 2011).

#### 2.8.2 Community Structure Spatial Consistency

For each participant, community structure was calculated using the Partition Stability algorithm (Delvenne et al. 2010). The categorical community structure maps were compared to an a priori template of the BGN using scaled inclusivity (SI). The SI method is a statistic that quantifies the spatial alignment between brain network communities to a referent template (Steen et al. 2011). The resulting SI maps were statistically compared between tapping tasks using the paired‐sample Jaccard index permutation statistic (Simpson et al. 2013).

### 2.9 Atlas‐Based Preprocessing

The structural T1 images were coregistered to the Colin 27 brain template (Schmahmann et al. 1999). The images were segmented to remove skull tissue and to segregate between WM, GM, and CSF. The T1 images were masked to include only GM and WM and were warped to the Colin template using Advanced Normalization Tools (ANTs) (Avants et al. 2011). The inverse of the resulting deformation map was applied to the Shen functional atlas (Shen et al. 2013), transforming the atlas to fit each participant's native anatomical image. The atlas was then resliced and coregistered to match each functional scan.

As with the voxel‐based preprocessing with functional images, the first 10 volumes were removed. Then, we performed slice‐time correction and realignment. A bandpass filter was applied at 0.009–0.08 Hz, and we averaged the signal from three segments (GM, WM, and CSF). The six motion parameters calculated during realignment were regressed out. The average signal was extracted from each of the 268 regions‐of‐interest (ROIs) of the Shen atlas to create the final time series.

### 2.10 Atlas‐Based Networks

#### 2.10.1 Network Generation

The functional atlas‐based brain networks were generated by applying Pearson's correlation between mean time series of all pairwise combinations of the 268 ROIs from the Shen atlas, yielding a 268 × 268 matrix. The BGN used for the voxel‐wise analyses is one of eight nonoverlapping a priori subnetworks that cover the entire brain. These subnetworks were translated to the Shen atlas, assigning each node of the Shen atlas to one of the eight subnetworks based on overlap. The BGN for the Shen atlas contained 45 nodes. The full list of these nodes can be found in Table S5.

#### 2.10.2 Mixed‐Effects Multivariate Regression Analysis

We used a mixed‐effects multivariate regression framework implemented in the MATLAB toolbox WFU_MMNET (Bahrami et al. 2018; https://www.nitrc.org/projects/wfu_mmnet), which utilizes Brain Connectivity Toolbox (BCT, Rubinov and Sporns 2010, https://www.nitrc.org/projects/bct/) to compute our two selected topological network features—global efficiency (GE) and clustering coefficient (CC). GE is a measure of network integration, while CC is a measure of network segregation. GE was computed at each node, by averaging the inverse of the shortest path length, or the shortest number of links, between each pairwise node of a network, representing the efficiency of information flow within the network (Bullmore and Sporns 2009). We averaged the value of each node to obtain a measure for GE across the whole brain. CC serves as a surrogate for local efficiency and is defined as the number of existing edges between node *i* and its immediate neighbor nodes, divided by the maximum possible edges with neighbor nodes (Watts and Strogatz 1998; Latora and Marchiori 2001). The balance between integration (GE) and segregation (CC) is crucial for optimizing neural interactions taking place at multiple levels in complex systems like the brain. Together, the two network measures for GE (global communication) and CC (local communication) measure how efficiently information is exchanged over the network (Tononi et al. 1994; Bullmore and Sporns 2009; Sporns, 2013; Fox and Friston 2012; Telesford et al. 2011; Simpson and Laurienti 2015).

We tested our hypotheses that changes in GE and CC in the BGN are associated with the experimental tasks, while controlling for confounding effects. We examined if and how our grouping covariate separating the self and sync tasks was associated with the brain connectivity and topology (GE and CC) in the BGN (local level) and all other brain regions combined (global level). More specifically, using this toolbox, the grouping covariate (tasks) as our covariate of interest (COI), confounding covariates (age, sex, years of education, and right‐handedness score), network metrics (GE and CC), and interactions of COI and network metrics were all used as independent variables. They were related to the probability (presence/absence) and strength of present connections (as outcome variables) in a two‐part regression model (Part I: Probability, Part II: Strength). The estimates and *p*‐values obtained for the interaction covariates allowed examining if/how the relationship between brain connectivity (probability/strength) and network metrics was modified by our grouping covariate. Significant differences in the BGN's connectivity and topology between the self and sync tasks were then examined by applying appropriate contrast statements on estimated residuals of interaction covariates in post hoc analyses. For more detail on contrast statements within this model, see Bahrami et al. (2022).

## 3 Results

### 3.1 Behavioral Tapping Data

Tapping performance on our tasks is shown in Table 2. Our paired‐sample *t*‐test that compared the absolute period error (ms) between the self task (*M* = 158.06, *SD* = 65.05) and the sync task (*M* = 18.59, *SD* = 6.74%) was significant, *t*(13) = 7.97, *p* < 0.001. The standard deviation (ms) of the absolute period error in the self task (*SD* = 65.05) was higher, reflecting higher tapping variability, while standard deviation was lower in the sync task (*SD* = 6.74), reflecting lower tapping variability. Synchronization error (ms) was negative (*M* = –14.38, *SD* = 8.01) in the sync task and for all participants, indicating that tap responses occurred on average before each tone onset.

**TABLE 2:** Finger tapping metrics for the self‐paced task and the 1‐Hz tone synchronized task.

Finger tapping metric	Self‐paced	1‐Hz tone sync	*p*‐value
Absolute period error (ms)	158.06 ± 65.05	18.59 ± 6.74	<0.0001
Synchronization error (ms)	—	−14.38 ± 8.01	—

*Note*: Values reported in this table are formatted as mean ± SD.

### 3.2 BGN Consistency

**FIGURE 1:**
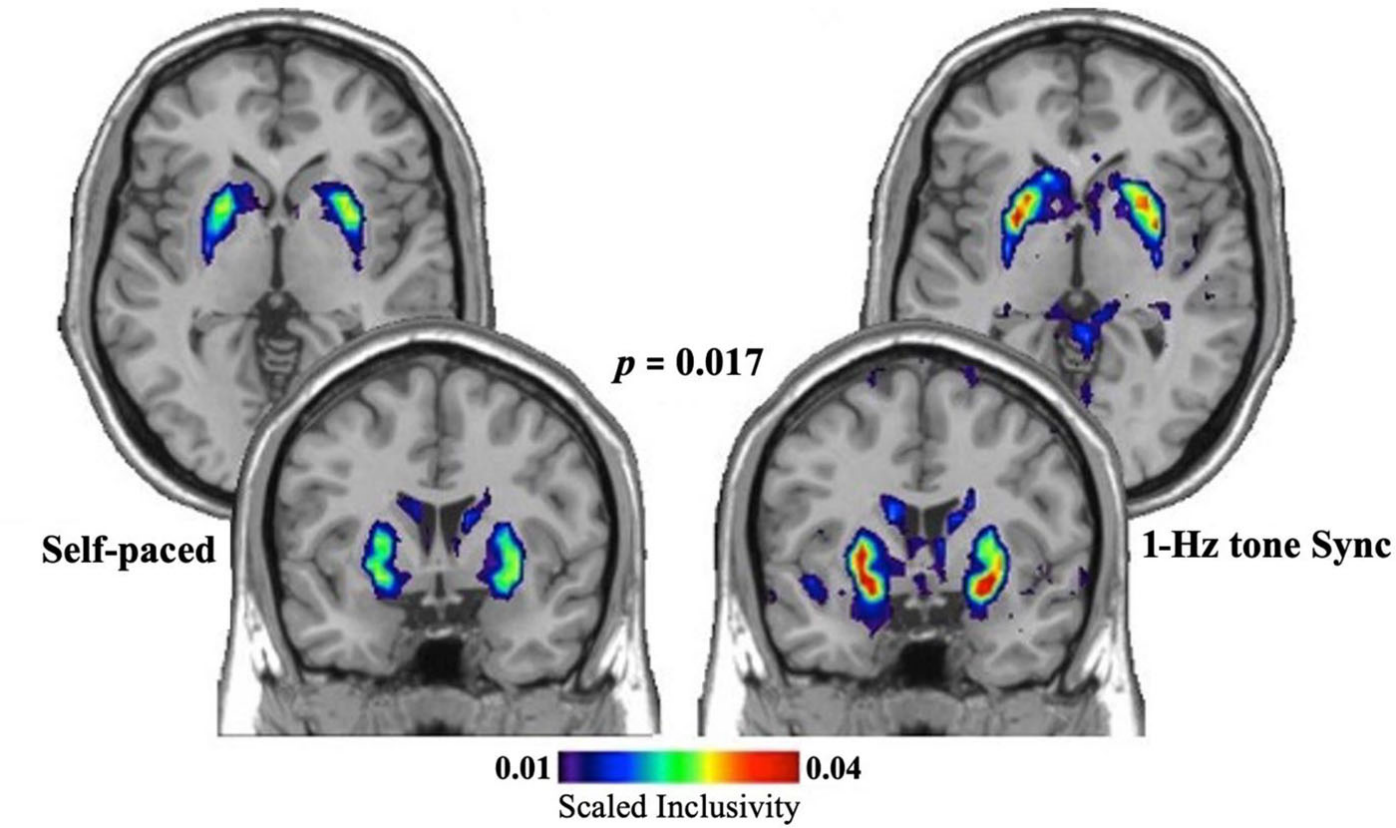
The consistency of the basal ganglia network (BGN) community in the self‐paced and the 1‐Hz tone synchronized tasks as evaluated by scaled inclusivity. The heat map represents the spatial overlap of community structure, demonstrating significantly higher and more consistent community structure alignment of the BGN in the sync task compared to the self task.

SI is a network topology metric that determines the consistency of network neighborhoods. Permutation tests performed on the SI metric revealed statistically greater consistency for the BGN community in the synchronized task compared to the self‐paced task with *p* = 0.017. Figure 1 clearly shows greater consistency of the BGN when the participants were tapping to the 1‐Hz tones rather than self‐pacing their taps.

### 3.3 Mixed‐Effects Regression Analysis

**FIGURE 2:**
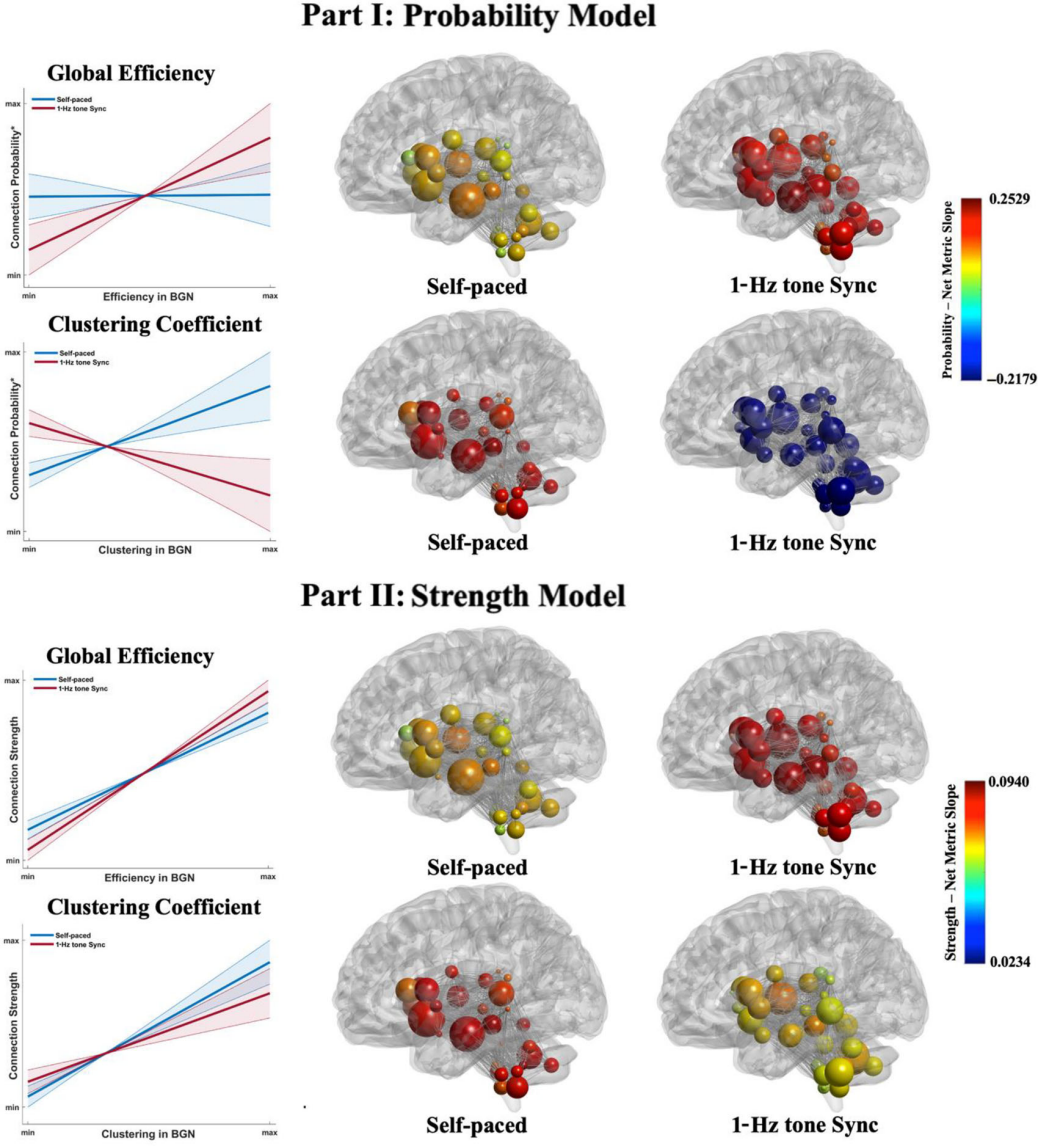
Visualization of significant results from our statistical analyses shown in Table 3 for the probability (Part I) and strength (Part II) models. The line plots (along with 95% confidence intervals) in this figure were created using the coefficients from our mixed model methodology to better illustrate the differences in the BGN. The line plots show how the probability and strength of brain connections change from their min to max values as the network metrics (global efficiency/clustering coefficient) change from their min to max values. Part I: Probability Model. In the synchronized task, regions with higher global efficiency (GE) within the basal ganglia network (BGN) are more connected (higher probability) and regions with higher clustering coefficient (CC) are less connected (lower probability) when compared to similar connections in the self‐paced task. The *y*‐axis in this figure is the log odds of connection probability, but the axis is labeled as connection probability for simplicity. To see the *x*‐ and *y*‐axis ranges (i.e., normalized GE and CC and predicted probability and log‐odds), see Figure S1. Part II: Strength Model. In the synchronized task, regions with higher GE within the BGN have stronger connections and regions with higher CC have weaker connections when compared to similar connections in the self‐paced task. For better visualization and comparison purposes, nodes are colored by the sum of their connection probability/strength slopes using the same color scale. Also, nodes are sized by their actual GE (Part I) and CC (Part II). To see the *x*‐ and *y*‐axis ranges, see Figure S1.

Table 3 presents the main findings of our statistical analyses using WFU_MMNET. See Tables S1–S5 and Figures S1 and S2 for the full results. All reported *p*‐values were corrected for multiple comparisons using the adaptive FDR procedure (Benjamini and Hochberg 2000). The significant differences are shown by line plots and visualized in brain space in Figure 2. Our data revealed that in the sync task, regions with higher efficiency (GE) in the BGN established more and stronger connections with each other as shown by the results from the probability (*β* = 0.180; *p* = 0.011) and strength (*β* = 0.024; *p* = 0.006) models. Corrected *p*‐values below 0.05 were considered statistically significant.

**TABLE 3:** Main findings of our mixed‐effects multivariate regression analyses using WFU_MMNET comparing the auditory–motor synchronized task over the self‐paced task in the basal ganglia network.

Model	Network parameter	Estimate	Pr > ∣*t*∣
Probability	Global efficiency	0.1801	0.0112
	Clustering coefficient	−0.3513	<0.0001
Strength	Global efficiency	0.0236	0.0064
	Clustering coefficient	−0.0166	0.0487

*Note*: Results reported in this table were obtained from contrast statements in post hoc analyses. The *p*‐values were adjusted using the adaptive FDR method (Benjamini and Hochberg 2000).

Our results also indicated a significant difference in the BGN's CC when comparing the two tasks. As Figure 2 and Table 3 show, in the 1‐Hz tone sync task, the regions with higher CC within the BGN are fewer and more weakly connected as shown by our probability (*β* = −0.351; *p* < 0.001) and strength (*β* = −0.017; *p* = 0.049) models. This indicated a shift toward a less clustered and more efficient BGN as suggested by changes in the CC and GE.

### 3.4 BGN Topology and Tapping Performance

To further examine the change in the BGN's topology between the sync and self tasks, we did additional analyses in which we examined if/how the tapping accuracy, defined by the absolute period error as our COI, is associated with the BGN's GE and CC in each group. We used WFU_MMNET and post hoc analyses to examine the relationships.

**FIGURE 3:**
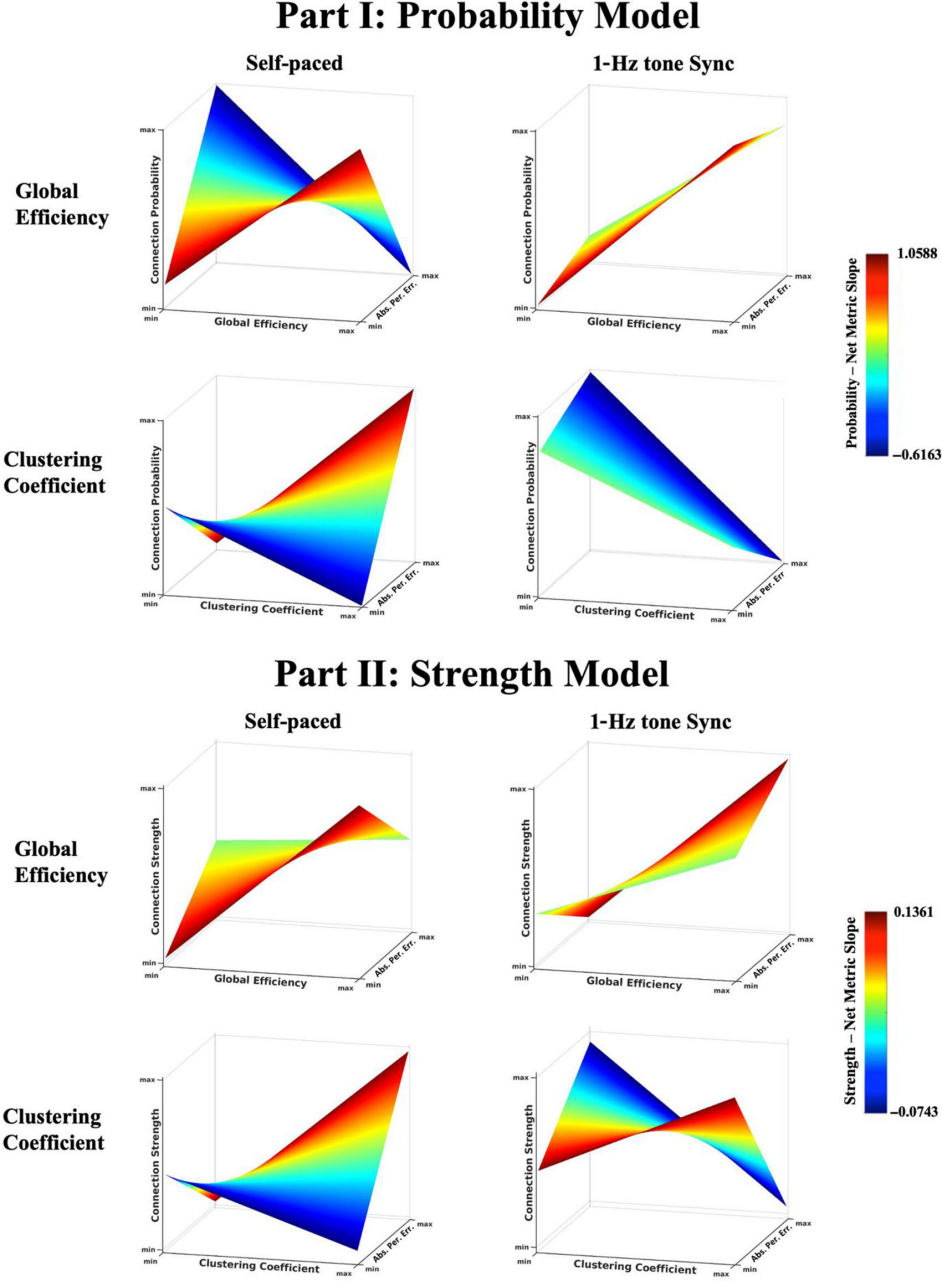
Visualization of the associations between tapping accuracy as indicated by absolute period error and the basal ganglia network (BGN)’s topology for the probability (Part I) and strength (Part II) models. The surface plots show how the relationship of connection probability (Part I) and strength (Part II) with network metrics for global efficiency (GE) and clustering coefficient (CC) is associated with the absolute period error in each task. The surface plots are colored by the slope of connection probability/network metrics (Part I) and connection strength/network metrics (Part II). Regions with higher GE become more weakly connected as the absolute period error increases (worse performance) in both the synchronized and self‐paced tasks, but with sync showing significantly smaller change. For CC, a reverse pattern is observed in the self task, with immediate neighbor nodes becoming more connected as the absolute period error increases, while in the sync task, the same pattern as for the GE (of the self task in the strength model) is seen. To see the *x*‐ and *y*‐axis ranges (i.e., normalized GE and CC and predicted probability and log‐odds) please see Figure S2.

Figure 3 and Table 4 show the mixed‐model regression results that combine the network topology data with tapping accuracy. In the self‐paced task, as the absolute period error increased (worse performance), the relationship between GE and connection probability significantly decreased (*β* = −0.311; *p* < 0.001), and, similarly, the relationship between GE and connection strength also significantly decreased (*β* = −0.032; *p* < 0.001). In other words, as performance became worse, the higher efficient regions within the BGN became less probably connected, and those that were connected had weaker connections. Then, the relationship between CC and connection probability significantly increased (*β* = 0.442; *p* < 0.001), and the relationship between CC and connection strength also significantly increased (*β* = 0.052; *p* < 0.001).

**TABLE 4:** Associations between tapping accuracy and the basal ganglia network (BGN) topology in the self‐paced task and the synchronized task based on the probability (Part I) and strength (Part II) models of our mixed‐model regression analyses.

Part I: Probability model		
Labels	Estimate	Pr > |*t*|
Global efficiency within BGN	−0.3108	<0.0001
Clustering coefficient within BGN	0.4422	<0.0001

Labels	Estimate	Pr > |*t*|
Global efficiency within BGN	−0.01875	0.6971
Clustering coefficient within BGN	−0.05063	0.3297

Part II: Strength model		
Labels	Estimate	Pr > |*t*|
Global efficiency within BGN	−0.03187	<0.0001
Clustering coefficient within BGN	0.05220	<0.0001

Labels	Estimate	Pr > |*t*|
Global efficiency within BGN	0.02876	<0.0001
Clustering coefficient within BGN	−0.03051	<0.0001

The absolute period error was lower in the sync task, and this task performance had less effect on the network topology. In the sync task, as the absolute period error increased (worse performance), the relationship between GE and connection probability slightly decreased, although not significantly (*β* = −0.019; *p* = 0.697), and the relationship between GE and connection strength significantly increased (*β* = 0.029; *p* < 0.001) within the BGN. The relationship between CC and connection probability decreased, although not significantly (*β* = −0.051; *p* = 0.330), and the relationship between CC and connection strength decreased significantly (*β* = −0.031; *p* < 0.001) within the BGN.

## 4 Discussion

We investigated the effects of AMS in healthy young adults using brain network science methods. Given the known effects of AMS on the BGN from a prior PET investigation (Koshimori et al. 2019), we focused this functional network investigation on the BGN. We confirmed our hypotheses that AMS is associated with changes in the intrinsic connectivity of the BGN. Specifically, we found significantly greater network consistency within the BGN community when participants synchronized their finger taps to 1‐Hz tones. In other words, the topology of the BGN community was more similar within the group in the sync condition (with AMS) compared to the self‐paced condition (no AMS). This topological similarity associated with AMS is indicative of increased functional connectivity within the BGN and suggests alteration of information flow in this important deep GM structure (Guimerà and Amaral 2005).

Our mixed‐model multivariate techniques further support the changes in functional connectivity in the BGN associated with AMS. Our results showed more and stronger connections between highly efficient nodes in the BGN while tapping to 1‐Hz tones, which indicates that the BGN was a more unified and synchronized functional unit with AMS. Taking this a step further, we performed multivariate regression analyses using the mixed model to incorporate tapping performance. In general, AMS was associated with lower absolute period error (i.e., better performance), reflecting higher tapping accuracy. When regressed with the imaging data, there was little change in BGN network topology with varying tapping performance in the sync task. However, interestingly, in the self‐paced task, as performance improved, the BGN topology increasingly resembled the networks of the sync task. This suggests that an improved internal rhythmic clock, such as the ability to keep a steady pulse without external cues, may be associated with enhanced BG synchronization. In other words, our BG findings could be related to either the AMS itself or just be related to more consistent auditory and/or motor timing. Future studies should consider these possibilities, such as presenting less predictable “jittered” AMS tasks.

In our data, the increased GE and decreased CC while syncing taps to 1‐Hz tones suggest a network topology of a highly integrated network rather than that of a segregated one. Arguably, with the 1‐Hz tones, the BGN became more in sync with itself and was more efficient at communicating with the rest of the brain, whereas in the self‐paced task, the BGN was more isolated, less synchronized.

Our mixed‐effects regression framework is a new method that allows for the statistical comparison of complex brain networks. We did not isolate the BGN for its analysis, such as placing a mask on it; rather, we analyzed it as a subnetwork or a subregion within the context of the whole‐brain network statistics. We discovered changes in GE and CC associated with our experimental tasks within the BGN. The efficiency of the BGN between higher degree nodes (high degree *k*, or number of links) significantly increased when tapping with AMS. On the other hand, immediate neighbor nodes significantly decreased their connectivity (decreased CC). With no AMS, connectivity patterns were reversed as connections between nodes of higher degree were less probable and less strong, and neighboring nodes developed more probable and stronger cluster connections.

Taken together, our analyses revealed that AMS increased the efficiency of the BGN to communicate within itself and with the rest of the brain. We believe that the increased network efficiency associated with AMS within the BGN builds upon the positive modulation observed at the neurochemical level in the motor DA pathways (Koshimori and Thaut 2018). With AMS, the continuous isochronous tones served as an external timekeeper, guiding participants to tap slightly prior to the next tone, in anticipation. Thus, our sync task tapping data revealed a negative synchronization error for all participants. The temporal template created by the predictable re‐occurrence of 1‐Hz tones facilitated the planning of the next movements (Thaut, Tian et al. 1998; Thaut et al. 2015). Such anticipation is a key component of AMS. Moreover, we suspect that the self‐paced task engaged additional cognitive resources related to the working memory network, as participants had to rely on an internal representation of the auditory template (Jantsen et al. 2007), as well as neural resources for explicit timing (Rao et al. 1997). Arguably, the self‐paced task is more cognitively demanding than the sync task.

Tapping performance was significantly better with rhythmic auditory cueing as demonstrated with lower absolute period error and variability at the group level. Furthermore, as previously discussed, the connectivity patterns observed in the self‐paced task associated with lower absolute period error (higher tapping accuracy) resembled those found in the sync task. In our sample, we controlled for musical training, as highly trained musicians are known to perform finger tapping tasks with lower anticipation errors and variability (Repp and Doggett 2007). When listening to naturalistic music, musicians tend to engage neural networks associated with actions, whereas nonmusicians tend to engage neural networks associated with perception (Alluri et al. 2017).

Our BGN findings are especially relevant to patients with PD. PD is a highly prevalent neurodegenerative disease (PHAC, 2018) that is characterized by motor and nonmotor symptoms resulting from the degeneration of DA cells in the BG. Motor symptoms include rigidity, tremor, bradykinesia (slowness of movement), and postural instability, while nonmotor symptoms include pain, mood disorders, sleep problems, cognitive impairment, or dementia (Kandel et al. 2021; Lim and Lang 2010; Wong et al. 2014). AMS is effective in remediating the characteristic shuffled gait and reducing freezing of gait in PD patients. As their stepping cadence entrains to the recurring auditory beat interval, their risk of falls decreases (Ghai, Ghai, Schmitz et al. 2018; Braun et al. 2019). Hence, AMS facilitates parkinsonian gait by improving velocity, cadence, and stride length, as demonstrated with PD patients “on” and “off” medication (McIntosh et al. 1997). Patients replicated their fastest stepping cadence without AMS even 24 h after their last RAS training session (Thaut et al. 1996). The RAS technique that builds upon AMS is now considered a potential nonpharmacological and neuromodulatory therapeutic intervention to remediate PD symptoms (Koshimori and Thaut 2023).

While no task‐based fMRI brain network analyses have been published to our knowledge, a resting‐state functional MRI (rs‐fMRI) connectivity analysis has been performed within the BGN using independent component analysis. Data were obtained from 90 patients with PD “on” and “off” medication and 19 age‐ and sex‐matched controls (Szewczyk‐Krolikowski et al. 2014). In the PD “off” compared to the “on” medication group, reduced connectivity within nine BGN clusters was observed, including the putamen and caudate bilaterally and anterior parts of the thalamus. The PD “on” group did not significantly differ from healthy controls. Our work builds on this “resting‐state” work and directly measures the effects of AMS on the BGN connectivity. Further work is necessary to determine if our BG findings in healthy individuals translate to PD patients.

### 4.1 Limitations

This study is not without limitations. As a preliminary investigation, our sample size, while large enough to obtain statistically significant findings, is too small to conclude generalizability of the findings. Additional investigations with larger sample sizes should be performed to determine reproducibility of our findings. Another limitation is that we had to use an atlas‐based analysis in addition to our voxel‐wise analyses. To perform our mixed‐model analyses to statistically compare network topologies between groups, especially with the addition of covariates, the model could not run with the large number of nodes in a voxel‐wise analysis. So, an atlas was used to obtain a smaller number of nodes for the mixed‐model regression to be performed. Interestingly, we obtained positive and complementary BG functional connectivity results from both the voxel‐wise and atlas‐based analyses. Lastly, we did not have a listening to isochronous tones control condition. Therefore, we did not evaluate the isolated effects of rhythmic auditory processing on the BGN, and this relevant question should be addressed in future studies.

## 5 Conclusion

This study was designed to uncover the neural mechanisms underlying the efficacy of rhythmic auditory cueing for motor tasks as a key tool for motor rehabilitation in people with neurological disorders. Our study demonstrated that AMS altered the patterns of functional brain connectivity within the BGN in healthy young adults, with AMS leading to a more synchronized BGN acting as part of a more integrated network, compared to a more segregated network without AMS. This study has clearly demonstrated the need to further investigate the functional brain networks of AMS in the PD population using network science methods to determine whether these findings underlie the success of AMS in the treatment of PD.

## Author Contributions

**Stéphanie K. Lavigne**: conceptualization, data curation, formal analysis, funding acquisition, investigation, project administration, validation, visualization, writing–original draft, writing–review and editing. **Johnathan Burdette**: conceptualization, funding acquisition, investigation, methodology, project administration, resources, supervision, writing–review and editing. **Mohsen Bahrami**: formal analysis, methodology, software, visualization, writing–review and editing. **Paul Laurienti**: conceptualization, methodology, resources, supervision, writing–review and editing. **Robert G Lyday**: data curation, software, writing–review and editing. **Michael H. Thaut**: conceptualization, formal analysis, resources, supervision, writing–review and editing.

## Ethics Statement

The study protocol was reviewed and approved by the WFUSM IRB.

## Conflicts of Interest

The authors declare no conflicts of interest.

## Peer Review

The peer review history for this article is available at https://publons.com/publon/10.1002/brb3.70695

## Supporting information




**Supplementary Material**: brb370695‐sup‐0001‐SuppMat.docx

## Data Availability

The data used in this study may be provided upon request. Codes are available upon request.
