# Supplementary material for: Altered Basal Ganglia Network Topology Associated With Auditory–Motor Synchronization
Source: Brain Behav. 2025 Aug 27;15(8):e70695. doi: 10.1002/brb3.70695 (PMC12381958; doi:10.1002/brb3.70695)
Supplement: Supplementary file 1 — Supplementary Material: brb370695‐sup‐0001‐SuppMat.docx [file BRB3-15-e70695-s001.docx]

Supplementary Material for

Altered Basal Ganglia Network Topology Associated with Auditory-Motor Synchronization

Stéphanie K. Lavigne^1,3^, Jonathan H. Burdette^2^, Mohsen Bahrami^2^, Paul J. Laurienti^2^, Robert G. Lyday^2^, Michael H. Thaut^1,3^

^1^Music and Health Science Research Collaboratory, Faculty of Music, University of Toronto, Toronto, Ontario, Canada

^2^Laboratory for Complex Brain Networks, Department of Radiology, Wake Forest University School of Medicine, Winston-Salem, North Carolina, United States of America

^3^Collaborative Program in Neuroscience, Faculty of Medicine, University of Toronto, Toronto, Ontario, Canada

**Table S1**

*Probability model: Solutions for fixed effects, global efficiency, and clustering coefficient considering the auditory-motor synchronization task over the self-paced task*

| **Effect** | **Sex** | **Estimate** | **Standard Error** | **DF** | ***t* Value** | **Pr > \|*t*\|** |
| --- | --- | --- | --- | --- | --- | --- |
| Intercept |  | -0.1291 | 0.07031 | 1000000 | -1.84 | 0.0664 |
| Distance |  | -0.3256 | 0.02059 | 1000000 | -15.81 | < .0001 |
| Distance2 |  | 0.1389 | 0.01467 | 1000000 | 9.47 | < .0001 |
| age |  | -0.0661 | 0.05279 | 1000000 | -1.25 | 0.2106 |
| education |  | 0.0386 | 0.05415 | 1000000 | 0.71 | 0.4759 |
| sex | f | -0.102 | 0.1173 | 1000000 | -0.87 | 0.3845 |
| sex | m | 0 | . | . | . | . |
| right_handedness |  | 0.02533 | 0.05662 | 1000000 | 0.45 | 0.6546 |
| task |  | -0.1323 | 0.06832 | 1000000 | -1.94 | 0.0528 |
| BGN |  | 0.4026 | 0.02074 | 1000000 | 19.41 | <.0001 |
| task*GE |  | -0.05614 | 0.04604 | 1000000 | -1.22 | 0.2227 |
| task*CC |  | 0.05959 | 0.04883 | 1000000 | 1.22 | 0.2224 |
| task*BGN |  | -0.04218 | 0.02844 | 1000000 | -1.48 | 0.138 |
| task*Distance2 |  | 0.0175 | 0.02335 | 1000000 | 0.75 | 0.4535 |
| task*Distance2 |  | 0.002143 | 0.01148 | 1000000 | 0.19 | 0.8518 |
| task*age |  | 0.07424 | 0.0513 | 1000000 | 1.45 | 0.1479 |
| task*education |  | -0.04935 | 0.05265 | 1000000 | -0.94 | 0.3486 |
| task*sex | f | 0.1893 | 0.114 | 1000000 | 1.66 | 0.0968 |
| task*sex | m | 0 | . | . | . | . |
| task*right_handedness |  | -0.05312 | 0.05504 | 1000000 | -0.97 | 0.3345 |
| GE |  | 0.2838 | 0.04056 | 1000000 | 7 | <.0001 |
| GE*BGN |  | -0.2698 | 0.03611 | 1000000 | -7.47 | <.0001 |
| GE*task*BGN |  | 0.2362 | 0.05556 | 1000000 | 4.25 | <.0001 |
| CC |  | -0.3024 | 0.03176 | 1000000 | -9.52 | <.0001 |
| CC*BGN |  | 0.496 | 0.03841 | 1000000 | 12.91 | <.0001 |
| CC*task*BGN |  | -0.4109 | 0.05771 | 1000000 | -7.12 | <.0001 |
| *Note.* GE = Global Efficiency; CC = Clustering Coefficient; BGN = Basal Ganglia Network; * = interaction between variables. Results from the probability model using WFU_MMNET (Bahrami et al., 2018) considering the BGN within its whole-brain context to compare the auditory-motor synchronized task over the self-paced motor task. The main hypothesized interactions are highlighted in grey (GE*task*BGN and CC*task*BGN). | | | | | | |

**Table S2**

*Strength model: Solutions for fixed effects, global efficiency, and clustering coefficient considering the auditory-motor synchronization over the self-paced task*

| **Effect** | **Sex** | **Estimate** | **Standard Error** | **DF** | ***t* Value** | **Pr > \|*t*\|** |
| --- | --- | --- | --- | --- | --- | --- |
| Intercept |  | 0.2121 | 0.006809 | 487000 | 31.15 | <.0001 |
| Distance |  | -0.05028 | 0.002371 | 487000 | -21.2 | <.0001 |
| Distance2 |  | 0.02996 | 0.001417 | 487000 | 21.14 | <.0001 |
| age |  | -0.00652 | 0.005095 | 487000 | -1.28 | 0.2004 |
| education |  | -0.0153 | 0.005214 | 487000 | -2.93 | 0.0034 |
| sex | f | 0.02219 | 0.01134 | 487000 | 1.96 | 0.0503 |
| sex | m | 0 | . | . | . | . |
| right_handedness |  | 0.009688 | 0.005461 | 487000 | 1.77 | 0.076 |
| task |  | 0.00225 | 0.0153 | 487000 | 0.15 | 0.8831 |
| BGN |  | 0.05316 | 0.002264 | 487000 | 23.48 | <.0001 |
| task*GE |  | 0.006613 | 0.006134 | 487000 | 1.08 | 0.281 |
| task*CC |  | -0.00512 | 0.005972 | 487000 | -0.86 | 0.3915 |
| task*BGN |  | 0.01651 | 0.003166 | 487000 | 5.21 | <.0001 |
| task*Distance |  | -0.00008 | 0.001622 | 487000 | -0.05 | 0.9611 |
| task*Distance2 |  | 0.000701 | 0.001002 | 487000 | 0.7 | 0.4839 |
| task*age |  | -0.00109 | 0.01144 | 487000 | -0.1 | 0.9243 |
| task*education |  | 0.008482 | 0.01172 | 487000 | 0.72 | 0.4692 |
| task*sex | f | -0.01901 | 0.02546 | 487000 | -0.75 | 0.4551 |
| task*sex | m | 0 | . | . | . | . |
| task*right_handedness |  | 0.01306 | 0.01228 | 487000 | 1.06 | 0.2874 |
| GE |  | 0.02991 | 0.003758 | 487000 | 7.96 | <.0001 |
| GE*BGN |  | 0.03488 | 0.00399 | 487000 | 8.74 | <.0001 |
| GE*task*BGN |  | 0.01698 | 0.006343 | 487000 | 2.68 | 0.0074 |
| CC |  | 0.06626 | 0.004486 | 487000 | 14.77 | <.0001 |
| CC*BGN |  | -0.02077 | 0.003832 | 487000 | -5.42 | <.0001 |
| CC*task*BGN |  | -0.01151 | 0.00619 | 487000 | -1.86 | 0.0629 |
| *Note.* GE = Global Efficiency; CC = Clustering Coefficient; BGN = Basal Ganglia Network; * = interaction between variables. Results from the strength model using WFU_MMNET (Bahrami et al., 2018) considering the BGN within its whole-brain context to compare the auditory-motor synchronized task over the self-paced task. The main hypothesized interactions are highlighted in grey (GE*task*BGN and CC*task*BGN). | | | | | | |

**Table S3**

*Mixed-effects multivariate regression model: Results obtained from contrast statements in post-hoc analyses*

| Model | Network Parameter | Estimate | Standard Error | DF | *t-*value | Pr > ∣*t*∣ |
| --- | --- | --- | --- | --- | --- | --- |
| Probability | Global Efficiency | 0.1801 | 0.07101 | 1000000 | 2.54 | .0112 |
|  | Clustering Coefficient | -0.3513 | 0.07455 | 1000000 | -4.71 | < .0001 |
| Strength | Global Efficiency | 0.02359 | 0.008648 | 487000 | 2.73 | .0064 |
|  | Clustering Coefficient | -0.01663 | 0.008435 | 487000 | -1.97 | .0487 |
| *Note.* Results reported in this table were obtained from contrast statements in post-hoc analyses. | | | | | | |

**
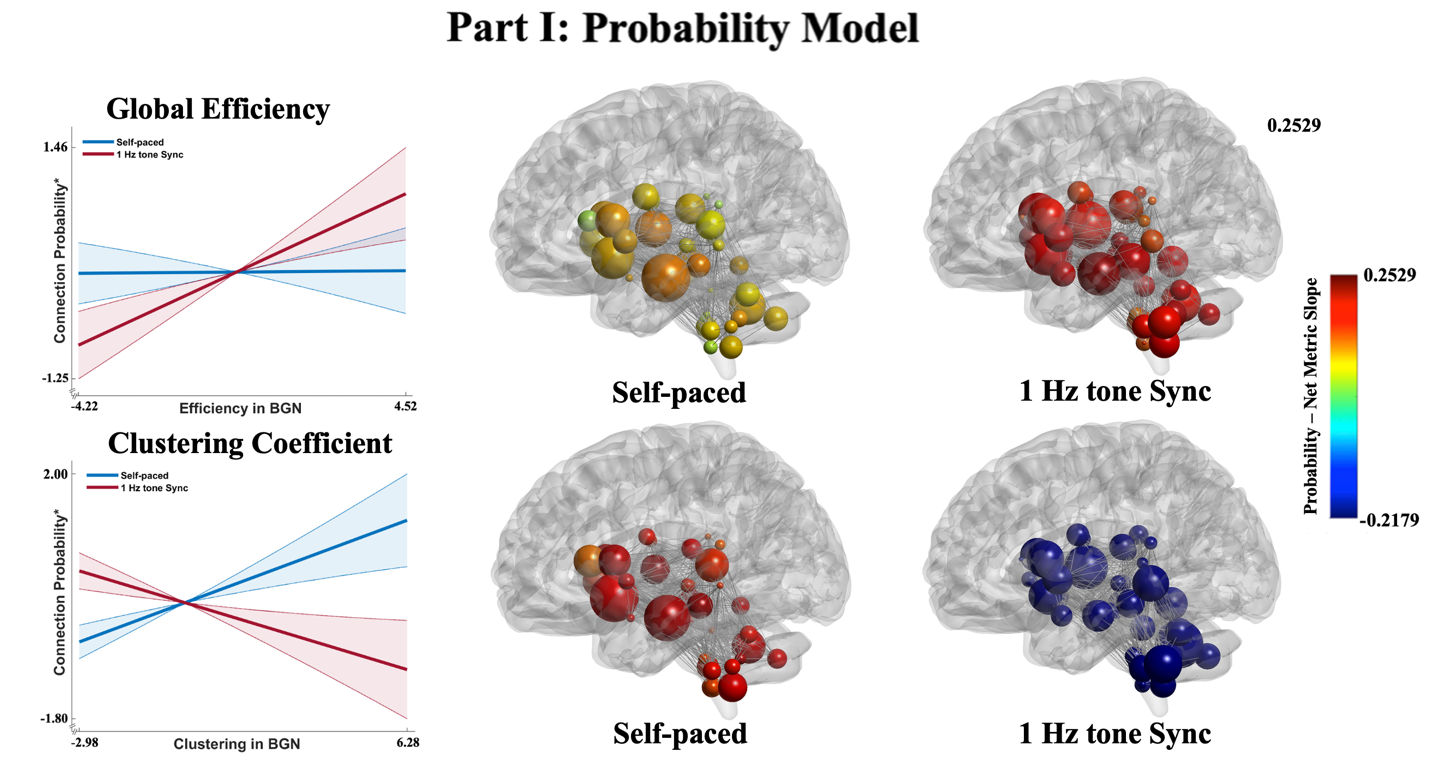
**

*
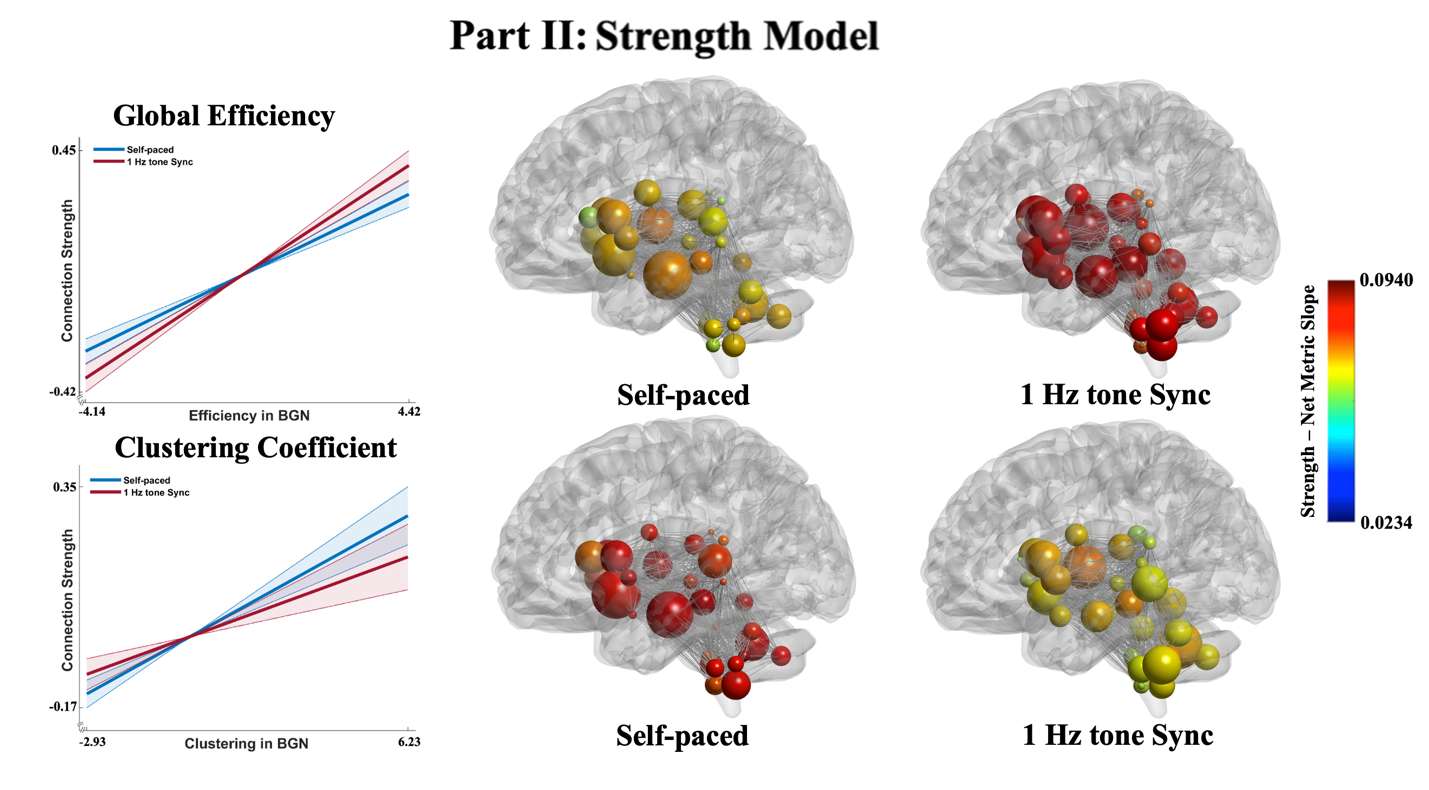
*

**Figure S1.** Visualization of significant results from our statistical analyses shown in Table 3 for the probability (Part I) and strength (Part II) models. The line plots (along with 95% confidence intervals) in this figure were created using the coefficients from our mixed model methodology to better illustrate the differences in the BGN. The line plots show how the probability and strength of brain connections change from their min to max values as the network metrics (global efficiency/clustering coefficient) change from their min to max values. Global efficiency (GE) and clustering coefficient (CC) and had raw ranges of [0.1669, 0.3229] and [0.0538, 0.3018] respectively, in our datasets. **Part I: Probability Model**. In the synchronized task, regions with higher GE within the basal ganglia network (BGN) are more connected (higher probability) and regions with higher CC are less connected (lower probability) when compared to similar connections in the self-paced task. The y-axis in this figure is the log odds of connection probability but the axis is labeled as connection probability for simplicity. **Part II: Strength Model**. In the synchronized task, regions with higher GE within the BGN have stronger connections and regions with higher CC have weaker connections when compared to similar connections in the self-paced task. For better visualization and comparison purposes, nodes are colored by the sum of their connection probability/strength slopes using the same color scale. Also, nodes are sized by their actual GE (Part I) and CC (Part II).

**Table S4**

*Associations between tapping accuracy and the basal ganglia network (BGN) topology in the self-paced task and the synchronized task based on the probability (Part I) and strength (Part II) models of our mixed-model regression analyses*

| **Part I: Probability Model**  **Self-paced** | | | | | |
| --- | --- | --- | --- | --- | --- |
| Labels | Estimate | Standard Error | DF | *t* Value | Pr > \|*t*\| |
| Global Efficiency within BGN | -0.3108 | 0.05195 | 501000 | -5.98 | < .0001 |
| Clustering Coefficient within BGN | 0.4422 | 0.05489 | 501000 | 8.06 | < .0001 |
| **1 Hz tone Sync** | | | | | |
| Labels | Estimate | Standard Error | DF | *t* Value | Pr > \|*t*\| |
| Global Efficiency within BGN | -0.01875 | 0.04816 | 501000 | -0.39 | .6971 |
| Clustering Coefficient within BGN | -0.05063 | 0.05193 | 501000 | -0.97 | .3297 |

**Part II: Strength Model**

| **Self-paced** | | | | | |
| --- | --- | --- | --- | --- | --- |
| Labels | Estimate | Standard Error | DF | *t* Value | Pr > \|*t*\| |
| Global Efficiency within BGN | -0.03187 | 0.005748 | 244000 | -5.54 | < .0001 |
| Clustering Coefficient within BGN | 0.05220 | 0.006737 | 244000 | 7.75 | < .0001 |
| **1 Hz tone Sync** | | | | | |
| Labels | Estimate | Standard Error | DF | *t* Value | Pr > \|*t*\| |
| Global Efficiency within BGN | 0.02876 | 0.006119 | 243000 | 4.70 | < .0001 |
| Clustering Coefficient within BGN | -0.03051 | 0.006582 | 243000 | -4.64 | < .0001 |


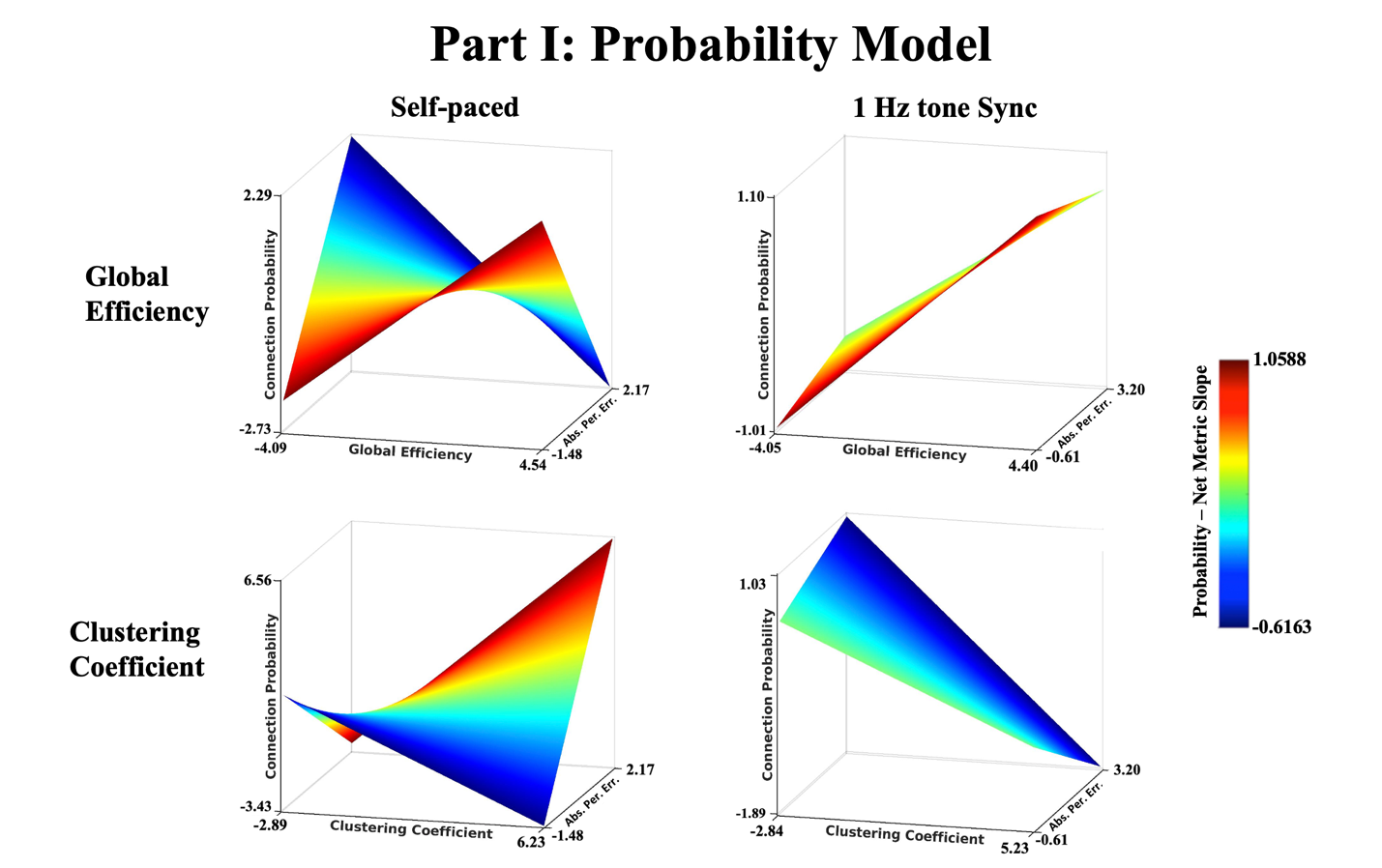


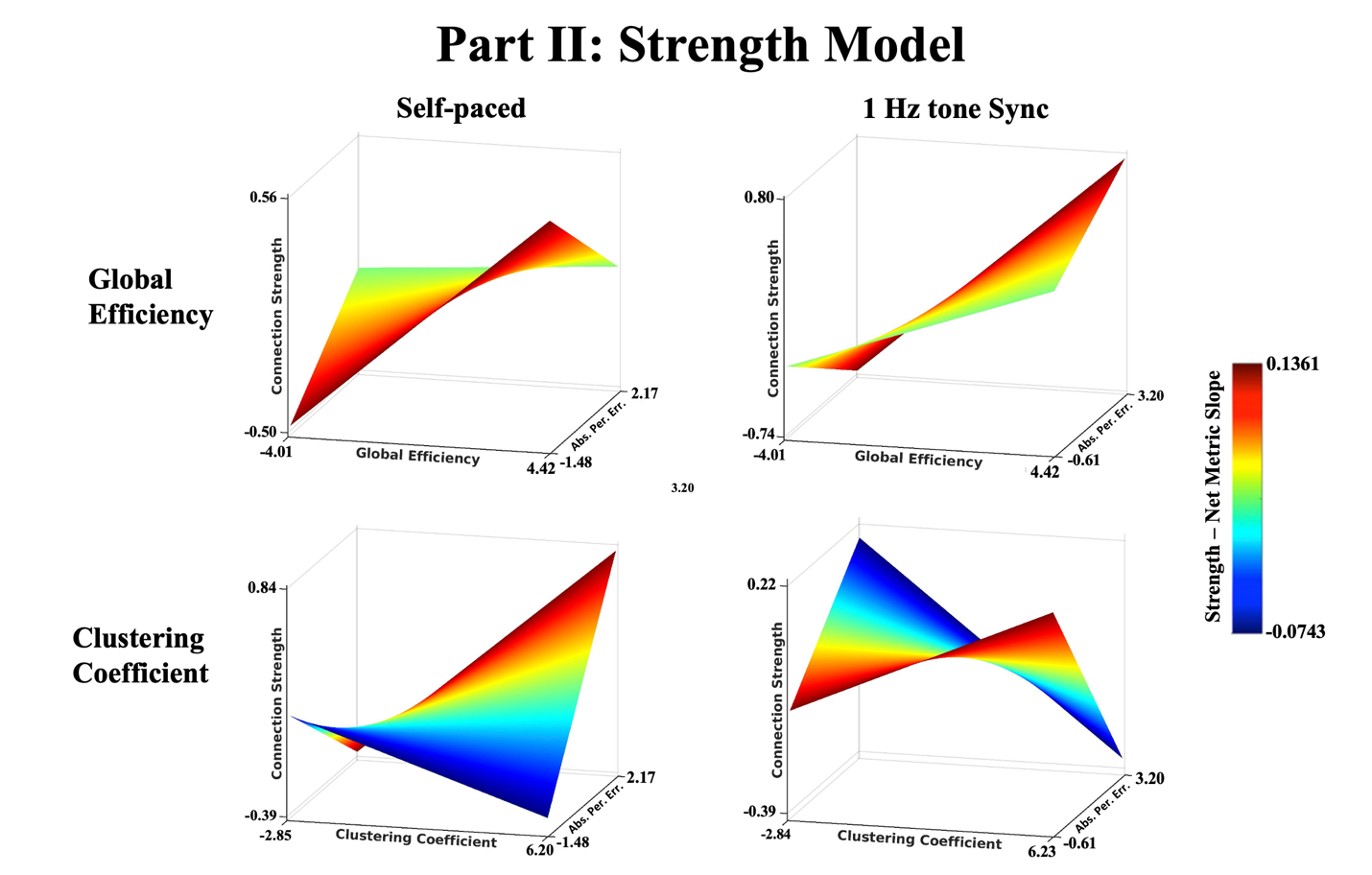


**Figure S2.** Visualization of the associations between tapping accuracy as indicated by absolute period error and the basal ganglia network (BGN)’s topology for the probability (Part I) and strength (Part II) models. The surface plots show how the relationship of connection probability (Part I) and strength (Part II) with network metrics for global efficiency (GE) and clustering coefficient (CC) are associated with the absolute period error in each task. The surface plots are colored by the slope of connection probability/network metrics (Part I) and connection strength/network metrics (Part II). Regions with higher GE become more weakly connected as the absolute period error increases (worse performance) in both the synchronized and self-paced tasks, but with sync showing significantly smaller change. For CC, a reverse pattern is observed in the self task, with immediate neighbour nodes becoming more connected as the absolute period error increases, while in the sync task, the same pattern as for the GE (of the self task in the strength model) is seen. GE and CC and had raw ranges of [0.1669, 0.3229] and [0.0538, 0.3018] respectively, in our datasets.

**Table S5**

*Nodes (n = 45) from the Shen atlas (2013) composing the basal ganglia network*

| Nodes included in the BGN |
| --- |
| 93 |
| 94 |
| 95 |
| 99 |
| 101 |
| 103 |
| 104 |
| 105 |
| 117 |
| 118 |
| 120 |
| 121 |
| 122 |
| 123 |
| 124 |
| 125 |
| 126 |
| 127 |
| 128 |
| 129 |
| 132 |
| 133 |
| 217 |
| 228 |
| 229 |
| 230 |
| 231 |
| 233 |
| 237 |
| 243 |
| 251 |
| 252 |
| 255 |
| 256 |
| 257 |
| 258 |
| 259 |
| 260 |
| 261 |
| 262 |
| 263 |
| 264 |
| 265 |
| 266 |
| 267 |

*Note.* The basal ganglia network (BGN) employed in our voxel-wise analyses is one of eight nonoverlapping a priori subnetworks that cover the entire brain. These subnetworks were translated to the Shen atlas (Shen et al., 2013), assigning each node of the Shen atlas to one of the eight subnetworks based on overlap. The BGN for the Shen atlas contained 45 nodes. This table presents the full list of these nodes.

**References**

Bahrami, M., Laurienti, P. J., & Simpson, S.L. (2018). A MATLAB toolbox for multivariate analysis of brain networks. *Human Brain Mapping, 40*(1), 175–186. <https://doi.org/10.1002/hbm.24363>

Shen, X., Tokoglu, F., Papademetris, X., & Constable, R. T. (2013). Groupwise whole-brain parcellation from resting-state fMRI data for network node identification. *NeuroImage, 82*, 403–415. <https://doi.org/10.1016/j.neuroimage.2013.05.081>
